# High-Sensitive Cardiospecific Troponins: The Role of Gender-Specific Concentration in the Diagnosis of Acute Coronary Syndrome (Descriptive Review)

**DOI:** 10.31083/j.rcm2407194

**Published:** 2023-07-06

**Authors:** Aleksey Chaulin

**Affiliations:** ^1^Department of Histology and Embryology, Department of Cardiology and Cardiovascular Surgery, Samara State Medical University, 443099 Samara, Samara Region, Russia; ^2^Research Institute of Cardiology, Samara State Medical University, 443099 Samara, Samara Region, Russia

**Keywords:** cardiospecific troponins, gender-specific concentrations, diagnostic threshold, 99 percentile, diagnosis, mechanisms of formation

## Abstract

Cardiospecific troponins are specifically localized in the troponin-tropomyosin complex and in the cytoplasm of cardiac myocytes. Cardiospecific troponin molecules are released from cardiac myocytes upon their death (irreversible damage in acute coronary syndrome) or reversible damage to cardiac myocytes, for example, during physical exertion or the influence of stress factors. Modern high-sensitive immunochemical methods for detecting cardiospecific troponins T and I are extremely sensitive to minimal reversible damage to cardiac myocytes. This makes it possible to detect damage to cardiac myocytes in the early stages of the pathogenesis of many extra-cardiac and cardiovascular diseases, including acute coronary syndrome. So, in 2021, the European Society of Cardiology approved diagnostic algorithms of acute coronary syndrome, which allow the diagnosis of acute coronary syndrome in the first 1–2 hours from the moment of admission of the patient to the emergency department. However, high-sensitive immunochemical methods for detecting cardiospecific troponins T and I may also be sensitive to physiological and biological factors, which are important to consider in order to establish a diagnostic threshold (99 percentile). One of the important biological factors that affects the 99 percentile levels of cardiospecific troponins T and I are gender characteristics. This article examines the role of gender-specific concentration of cardiospecific troponins in the diagnosis of acute coronary syndrome and the mechanisms of formation of gender-specific serum levels of cardiospecific troponins T and I.

## 1. Introduction

According to the statistics of the World Health Organization (WHO), acute coronary syndrome is one of the most dangerous forms of cardiovascular pathologies and occupies a leading place in the structure of mortality in most countries of the world [1]. According to the Eurasian Association of Cardiologists, the highest mortality rates of patients from acute coronary syndrome among European countries are recorded in Russia, Ukraine, Belarus, Bulgaria and Lithuania. Among the European countries, the hospital mortality rate ranges from 6 to 14%, while in Russia the mortality rate was 18.6% and 17.7% in 2015 and 2016, respectively [2, 3]. Prevalence of acute coronary syndrome is lower among women than among men. Thus, the proportion of women among patients with acute coronary syndrome, according to the Russian RECORD-3 register for 2015, was 39%. Among patients with myocardial infarction with and without ST elevation, women also constituted a minority—32% and 44%, respectively [3, 4].

Current Russian (Russian Society of Cardiology) [5, 6] and foreign (European Society of Cardiology, American Heart Association, American College of Cardiology) guidelines [7, 8] recommend the use of cardiospecific troponin tests as the “gold standard” for diagnosing myocardial infarction. This is due to their high cardiospecificity (localization only in cardiac myocytes) [9, 10], diagnostic and prognostic value in forecasting myocardial infarction in case of acute conditions and the risk of all-cause mortality and cardiovascular events in the general population [11, 12].

Due to differences in the prevalence of acute coronary syndrome and in the degree of increase in cardiospecific troponin levels in men and women at the early stages of diagnostics, several authors propose the gender-based approach to the early diagnostics of acute coronary syndrome [13, 14]. However, to date, this approach is not sufficiently covered in a number of guidelines and there are no specific recommendations for the diagnosis of acute coronary syndrome depending on the gender identity. This is largely due to the inconsistency of the results of clinical studies performed in this regard. In addition, the specific physiological mechanisms underlying the formation of gender-based variations in serum levels of cardiospecific troponins have not been established.

The purpose of this review is to systematize information on the importance of taking into account the data on the gender (sex) characteristics of the content (in the range of the 99th percentile) of cardiospecific troponins in the diagnostics and prognosis of development of the acute coronary syndrome, as well as on the possible mechanisms for formation of gender differences in the cardiospecific troponin content levels.

## 2. Cardiospecific Troponins: Biochemistry and Physiological Role

The cardiospecific troponin complex regulates striated muscle contraction and consists of three subunits: troponin C, T, and I, which are designated according to their functional significance. Troponin C (the calcium-binding subunit) binds to calcium ions, which initiates conformational changes in the cardiospecific troponin complex and tropomyosin, leading to the opening of myosin-binding sites on the actin molecule. Subsequently, the myosin head interacts with the myosin-binding sites, resulting in the formation of transverse (actin-myosin) bridges. The protein molecule troponin T is a tropomyosin binding subunit; it attaches two other troponin subunits (troponin C and troponin I) to actin filaments. The protein molecule troponin C is a calcium-binding subunit; it binds calcium ions that enter the cytoplasm during the systolic phase [9, 15, 16]. The importance of cardiospecific troponins in the regulation of myocardial contractile function is demonstrated by the fact that small changes in the amino acid sequence of the protein molecules cardiospecific troponin I, cardiospecific troponin T, and troponin C are associated with significant and life-threatening violations of the contractile function of the heart’s muscular layer, known as hereditary cardiomyopathies [9].

**Fig. 1.**
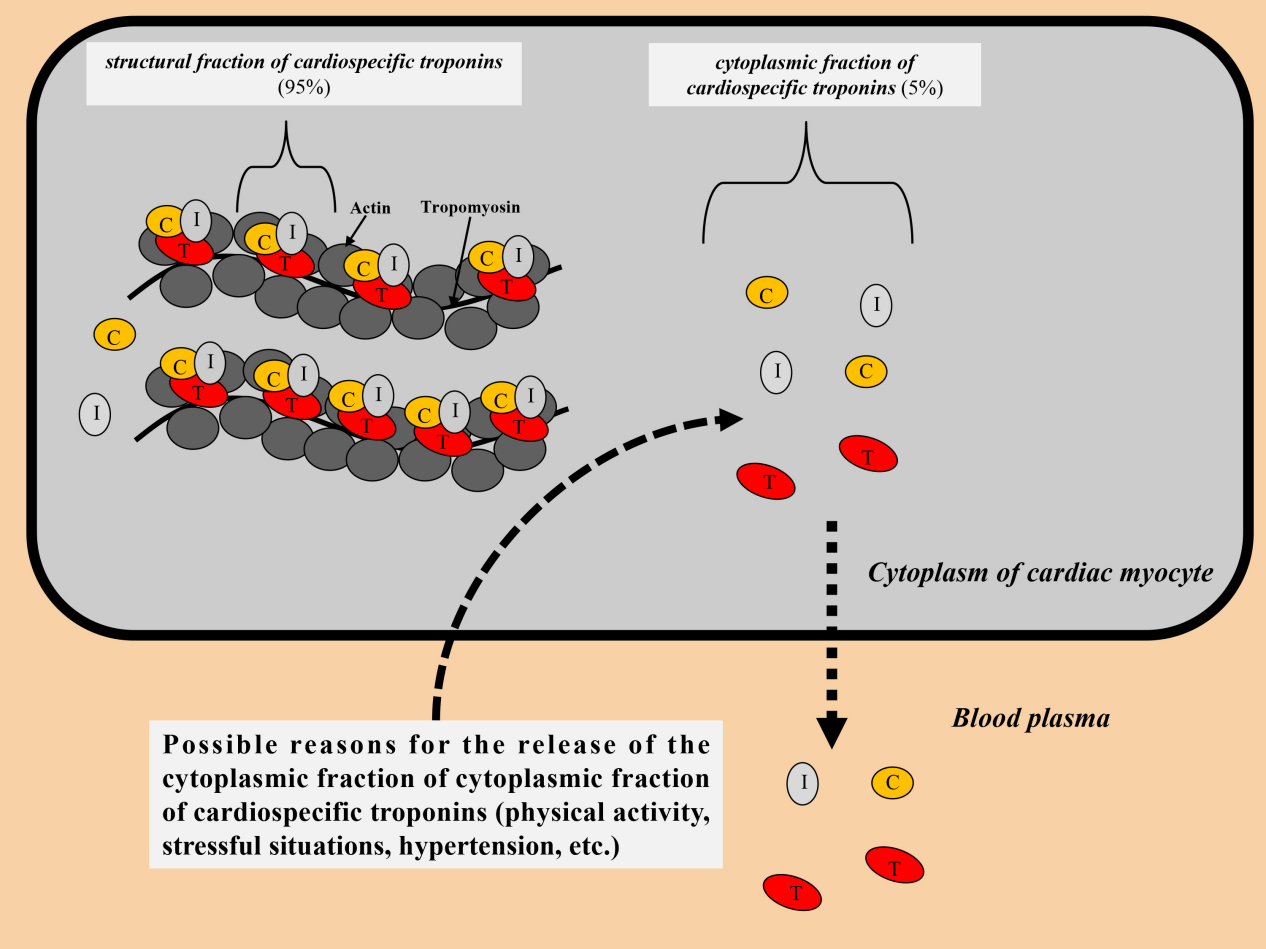
**Distribution of cardiospecific troponins in the cytoplasm of cardiac myocytes**. C, troponin C; I, troponin I; T, troponin T.

The most part of the cardiospecific troponins (approximately 90–95%) is associated with myofilaments, and a small concentration (5–10%) is in a free state in the cytosol (Fig. 1). After damage to the cell membrane of cardiac myocytes as a result of exposure to a number of adverse factors (for example, ischemia, inflammatory cytokines, cardiotoxic agents, etc.), cardiospecific troponin molecules are initially released from the cytoplasm of cardiac myocytes into the interstitium, and then into the general bloodstream. Protein molecules of troponin T and troponin I are localized in cardiac myocytes and skeletal myosymplasts, but they are encoded by different genes in each type of muscle, resulting in the formation of two different immunochemical products. Laboratory diagnostic studies of cardiovascular pathology, in particular myocardial infarction, are based on the use of high-affinity anti-troponin antibodies against cardiospecific troponin T and cardiospecific troponin I [16, 17, 18].

## 3. High-Sensitive Cardiospecific Troponins: Characteristics of Detection Methods and Important Analytical Parameters

Since Katus and colleagues developed the first immunotest to measure cardiospecific troponin T in 1991 [19], an extremely long journey has been made from the development to the introduction of high-sensitive troponin tests into current clinical practice [20, 21].

The use of high-sensitive cardiospecific troponin laboratory diagnostic tests is an important step forward due to their high sensitivity to cardiac myocytes damage, as they are able to determine serum cardiospecific troponin levels approximately 10–100 times lower than conventional tests, leading to more accurate and timely diagnostics [22, 23].

According to the International Federation of Clinical Chemistry (IFCC) guidelines, two criteria are used to define this new generation of (high-sensitive) cardiospecific troponin tests: (1) the coefficient of variation (CV) at the 99th percentile value should be 10% or less (the most optimal immunoassays), although tests with the error of more than 10 and 20% or less are still considered clinically acceptable; (2) the concentration of cardiospecific troponins should be above the minimum determinable concentration (limit of detection (LoD)) in more than 50% of healthy individuals [20, 24, 25].

New laboratory diagnostic tests allow to conduct earlier diagnostics and to quickly exclude myocardial infarction due to high sensitivity [26], however, the second key immunoassay criterion, specificity, is affected significantly. Clinically, this is expressed by the presence of a wide range of other troponin-positive non-cardiac and cardiac conditions other than myocardial infarction [27, 28]. Although not all mechanisms of elevation of cardiospecific troponin levels are known, in some conditions this may be due to a decrease in oxygen supply to the cardiac myocytes, and it is not clear whether to cardiac myocytes is always irreversible, which inevitably leads to cardiac myocytes necrosis, or whether some diseases can cause reversible damage. There may also be a false increase in cardiospecific troponins in the blood serum, which is not associated with damage to cardiac myocytes. Common causes of a false increase in cardiospecific troponins are: rheumatoid factor, heterophilic antibodies, alkaline phosphatase, macrotroponin, hemolysis, fibrin clots [29]. The main conditions (physiological and pathological) that cause an increase in the levels of cardiospecific troponins are shown in Table 1.

**Table 1.** **The main reasons of cardiac myocytes damage (physiological and pathological) and increased concentration of cardiospecific troponins**.

The nature of cardiac myocytes damage	Reasons
Myocardial damage caused by ischemia	Acute coronary syndrome/Myocardial infarction
Cardiac myocytes damage not caused by ischemia in cardiac pathologies	- Myocarditis, endocarditis, pericarditis;
	- The use of cardiotoxic drugs, for example, anthracyclines;
	- Radiofrequency or cryoablation therapy;
	- Cardiomyopathy and heart failure;
	- Pacing or defibrillation;
	- Infiltrative pathologies of the heart, for example, amyloidosis;
	- Takotsubo syndrome
Cardiac myocytes damage not caused by ischemia in extracardiac pathologies	- Sepsis;
	- Chronic renal failure;
	- Chronic obstructive pulmonary disease;
	- Pulmonary embolism;
	- Neurogenic diseases (stroke, subarachnoid hemorrhages);
	- Arterial hypertension
Physiological conditions	- Physical activity;
	- Stressful situations
Without cardiac myocytes damage (false positive factors)	- Heterophilic antibodies;
	- Alkaline phosphatase;
	- Hemolysis of the sample;
	- Rheumatoid factor;
	- Fibrin clots in the sample;
	- Cross-reaction of diagnostic antibodies with skeletal troponins

Currently, all available high-sensitive methods for determining cardiospecific troponins have a single diagnostic threshold value for acute coronary syndrome diagnostics, based on the value of the 99th percentile, which is calculated for a healthy population [25, 30]. However, this threshold value can vary significantly depending on the methodology for determining (manufacturer) cardiospecific troponins [30, 31]. According to the IFCC, the main manufacturers of high-sensitive immunochemical reagent kits for determining cardiospecific troponins are: Abbot (Austin, TX, USA), Beckman Coulter (Brea, CA, USA), bioMerieux (Marcy-l’Étoile, France), ET Healthcare (Palo Alto, CA, USA), LSI Medience (Tokyo, Japan), Fujirebio (Tokyo, Japan), Ortho Clinical Diagnostics (Raritan, New Jersey, USA), Quidel/Alere (San Diego, CA, USA), Roche (Basel, Switzerland), Siemens (Munich, Germany), etc. [30].

The additional important diagnostic advantage of high-sensitive tests for immunochemical determination of cardiospecific troponins is the ability to determine cardiac myocytes damage at the subclinical level, which can be used to monitor and assess the prognosis of patients suffering from a number of chronic pathologies, including coronary heart disease [32], during the treatment of oncological diseases by using chemotherapeutic compounds, which are characterized by cardiotoxicity [33], as well as chronic obstructive pulmonary disease [34], chronic kidney disease [35], diabetes mellitus (DM) [36], arterial hypertension [37], etc.

An important advantage of modern high-sensitive cardiospecific troponin immunotests is the ability to detect cardiospecific troponin molecules in noninvasively obtained biological material (oral fluid, urine, sweat) [38, 39, 40, 41, 42]. When receiving these biomaterials, a number of advantages can be noted: non-invasiveness, atraumatic, painless, no specially trained medical personnel is required, the ability to conduct preliminary diagnostics at home (test strips). Therefore, this method will allow for the diagnosis of diseases in a non-invasive way. However, to date, this is a little-studied and controversial area that requires further research to confirm these possibilities.

## 4. Gender Specificities of Cardiospecific Troponin Levels

Gender specificities of concentrations are characteristic of a number of laboratory analytes (red blood cells (RBC) count, hemoglobin, creatinine concentrations, etc.), which is widely used in modern clinical practice. As for cardiac markers, for the first time information about the gender specificities of laboratory tests was found during the study of creatine phosphokinase activity, the test for determining which was used to diagnose myocardial infarction in the 60–70ies of the XX century. It was found that healthy men had higher creatine phosphokinase activity than healthy women, and in dark-skinned people, creatine phosphokinase activity was higher than in Caucasians [43]. This was also characteristic of the muscle-brain (MB)-fraction of creatine phosphokinase, both for activity (u/L) and for the concentration of creatine phosphokinase-MB (creatine phosphokinase-MB mass), measured in ng/mL. The mechanism of these differences, according to academic specialists, was largely due to the differences in skeletal muscle mass in men compared to women [43]. Later, gender differences were noted for natriuretic peptides, and, according to the authors, they were due to the different influence of male and female sex hormones on the production of natriuretic peptides in the myocardium [44]. However, with the introduction of the first tests for immunochemical determination of cardiospecific troponins, gender features ceased to be determined, which was probably due to the low sensitivity of these test systems, because they determined troponin concentrations in only 5% of healthy subjects [22, 23, 24]. Therefore, at that time, the prevailing opinion was that cardiospecific troponins are strictly intracellular molecules that appear in the blood serum only in case of serious pathologies of the myocardium, and certain positive levels of cardiospecific troponins in patients with unconfirmed myocardial infarction were most often interpreted as false positive results. This opinion was also supported by the studies reporting a high prevalence of false positive results of cardiospecific troponins in patients with rhabdomyolysis in case of skeletal muscle pathologies [45, 46]. As the sensitivity of laboratory methods increased, cardiospecific troponin levels began to be determined in the blood of a larger number of healthy individuals (which allowed to regard cardiospecific troponin as the metabolic products of cardiac myocytes) and the first reports of possible gender-based variations in cardiospecific troponin levels appeared. Thus, Apple *et al*. [47], studying the reference limits of cardiospecific troponin levels in a large sample of patients (n = 686) using eight immunochemical determination tests, found the presence of gender variations in 2 methods of immunochemical determination of cardiospecific troponin I. At the same time, the average level of cardiospecific troponin I in men was 1.2–2.5 times higher than in women, according to the nonparametric statistical analysis of the results [47]. However, this study is actually the only one that reported any gender variations for the moderately sensitive research methods, and therefore this was not reflected in practical medicine. With the introduction of high-sensitive immunochemical tests, it has been shown that in 80% of healthy individuals, determinable cardiospecific troponin concentrations exceed the determination limit [48], and the rates are significantly higher in men than in women, leading to a more detailed study of the potential gender specific 99th percentile. In a large study including 524 healthy subjects (272 males, 252 females), 99th percentile levels were calculated for 19 cardiospecific troponin tests: one cardiospecific troponin T test by Roche and 18 cardiospecific troponin I tests by Abbott, Alere, Beckman, bioMerieux, Instrumentation Laboratory, Ortho-Clinical Diagnostics, Singulex, Siemens and Roche, of which five were analytically classified as high-sensitive. The study found that 99-th percentile levels exceeded the determination limit in 80% of people in case of high-sensitive immunochemical determination tests, while moderately sensitive tests determined measurable cardiospecific troponin levels in about 25% to 30% of patients. Gender specificities of the 99th percentile were typical of all high-sensitive test systems for determining cardiospecific troponin I, the values of which in men were 1.2–2.4 times higher than in women. Approximately similar values were demonstrated by the high-sensitive analysis for cardiospecific troponin T: the 99th percentile for men was 20 ng/L, and for women it was 13 ng/L, while the overall (regardless of gender) calculated 99th percentile was 15 ng/L. Besides, the gender-specific 99th percentile was characteristic of some moderately sensitive test systems, according to which cardiospecific troponin levels were 1.3–5 times higher in men than in women [48].

Saenger *et al*. [49] showed that statistically significant differences were observed in high-sensitivity cardiospecific troponin T concentrations in men and women, and the 99th percentile for healthy men was 1.7 times higher than for healthy women (15.5 ng/L versus 9.0 ng/L, respectively). In another larger study, Gore and colleagues noted similar results in three independent cohorts of patients in which high-sensitive cardiospecific troponin T concentrations were analyzed based on the age, sex, and race stratification [50]. It is important to note that more than 10% of men aged 65 to 74 years without cardiovascular pathology had high-sensitivity cardiospecific troponin T values above the threshold value (99th percentile) (>14 ng/L). The researchers noted that in each cohort, the level of the 99th percentile increased with age. In addition, healthy older men had a higher 99 percentile than healthy older women. The academic specialists also found significant differences in the threshold levels of high-sensitive cardiospecific troponin T depending on age and gender specificities: men (disregarding age) = 23 ng/L, men 50–64 years old = 28 ng/mL, men under 50 years old = 19 ng/L; women (disregarding age) = 9 ng/mL, women 50–64 years old = 14 ng/mL, women under 50 years old = 9 ng/mL [50]. Thus, gender and age must be taken into account when calculating the 99th percentile levels of high-sensitive cardiospecific troponins. Whereas the use of the single threshold value (14 ng/L) for high-sensitive cardiospecific troponin T analysis can lead to overdiagnosis of myocardial infarction, especially in men and the elderly, since their normal (baseline) level significantly exceeds the 99th percentile recommended by the immunotests manufacturer. The studies presented are indicative of the need for a close study of the gender-age specificities of cardiospecific troponins for clinical validation [50].

In the current European Society of Cardiology guidelines for the diagnostics and treatment of myocardial infarction without ST-segment elevation [51], the diagnosis of myocardial infarction is based not on the single value of the cardiospecific troponin, but on two main algorithms based on the dynamic changes in cTn at the 0 moment (on admission to the emergency care department and first blood test) and after 3 hours or after 1 hour. Only validated, high-sensitive cardiospecific troponin immunotests with confirmed threshold levels or cut-off values should be used to apply these algorithms. Notably, the 0/3 h algorithm makes a clear reference to the upper control limit of the 99th percentile, and this is also specified in the fourth universal definition of myocardial infarction [8], while the 0/1 h algorithm uses cutoffs below the 99-th percentile, calculated for specific cardiospecific troponin tests of immunochemical determination. The most important role in these diagnostic algorithms is played by the kinetics of the increase in cardiospecific troponin levels during the first hours from the moment of chest pain/admission to the emergency care department. The positive predictive value of these algorithms for patients with myocardial infarction, i.e., those who meet the “rule-in” criteria, is 75–80%. Some patients that meet the “rule-in” criterion with diagnoses other than myocardial infarction may have conditions (e.g., takotsubo cardiomyopathy, myocarditis, etc.) that usually require hospitalization and coronary angiography for accurate diagnosis [51]. As the upper control limit of the 99th percentile is not always gender-specific, and the 0/1 hour algorithm does not use gender-based cut-offs, the absence of specificity and relatively low positive predictive value of cardiospecific troponins in patients with myocardial infarction may be partly explained by the inadequate threshold value, which is equal for both men and women.

It is worth noting that many researchers have not yet come to a consensus on the feasibility of using a gender-specific 99th percentile as the diagnostic threshold [24, 51, 52, 53, 54, 55]. Its use can lead to an excess of patients with the elevated cardiospecific troponins level which is not associated with myocardial infarction [52, 53]. However, on the other hand, the application of general cut-offs in clinical practice may lead to underestimation of the risk of acute myocardial infarction, especially in female patients [54, 55]. Thus, Novak and colleagues [56] found that women constitute a high-risk group which receives less of the treatment methods for myocardial infarction recommended by the guidelines, especially less frequent use of secondary prevention of cardiovascular complications and rare cardiac catheterization. That is why determination of the threshold level of cardiospecific troponin in women is crucial, since an incorrect determination 99th percentile can lead to incorrect interpretation of the result and further management of these patients.

A group of researchers led by Trambas [57] found that the transition from a moderately sensitive method for determination cardiospecific troponin I to a high-sensitive analysis for cardiospecific troponin I significantly increased the number of patients with an increased concentration of cardiospecific troponin I, while no significant changes were found in men [57]. Introduction of gender-specific threshold reference values did not lead to an increase in the number of cases of myocardial infarction among the female patients. However, the use of a gender-specific 99 percentile allowed to identify those women who have a high risk of future cardiovascular complications [57]. Similar results were demonstrated in case of use of high-sensitivity cardiospecific troponin T in the study (study of bypass angiopasty revascularization in case of type 2 DM) [58]. Within this study they observed 684 women and 1601 men with type 2 DM and stable coronary artery disease for 5 years. The results showed that among patients with type 2 DM and stable coronary artery disease, women with circulating levels of high-sensitivity cardiospecific troponin T that are within the “normal” range (the commonly used 99th percentile disregarding gender) are at increased risk of serious cardiovascular complications, which exceeds the rates observed among men with similar levels of high-sensitive cardiospecific troponin T [58]. Thus, this study also shows the need to revise the 99th percentile with account taken of the gender.

## 5. Possible Mechanisms for Formation of Gender Specificities of Cardiospecific Troponins

Under physiological conditions, the most common causes of elevated cardiospecific troponins are physical activity and psychoemotional stress [59, 60, 61]. These physiological conditions can lead to myocardial overload, small-scale processes of cardiac myocytes apoptosis due to increased activity of the sympathoadrenal system, increased activity of prooxidant mechanisms, reversible damage to cardiac myocyte membranes, which is accompanied by the release of cytosolic cardiospecific troponin molecules, and a slight increase in serum concentrations of cardiospecific troponins [62, 63, 64, 65]. Thus, elevated levels of cardiospecific troponins in healthy individuals may reflect the response of cardiac myocytes to the influence of stress factors. However, in men and women, the activity of protective mechanisms of different cells, including the cardiac myocytes, against stress factors differs, which may be a possible explanation for gender differences in serum levels of cardiospecific troponins. Thus, the recent study [66] demonstrated that the levels of cardiospecific troponin T after the same physical activity in male athletes were significantly higher than in female athletes, which is indicative of the different response of cardiac myocytes to physical activity in men and women.

In addition to this, the study by Tiller *et al*. [67] also showed more pronounced disorders of physiology of the cardiovascular system in men than in women after an ultramarathon. Potentially, these negative effects could lead to a greater release of cardiospecific troponins in men than in women.

Schwarzenberger and colleagues have shown that men are less protected from damage to cardiac myocytes. This is evidenced by the fact that after heart surgery, men had a greater increase in the level of cardiospecific troponin in the blood serum than women [68]. That said, the groups of men and women were formed in accordance with the same characteristics (same body mass index, duration of artificial circulation, duration of aortic compression during surgery, etc.), which could potentially affect the degree of myocardial damage and the release of cardiospecific troponins. Thus, variations in damage and release of cardiospecific troponins, apparently, are due to the gender-based differences in the degree of ischemia-reperfusion injury of cardiac myocytes.

Gender differences in the degree of cardiac myocytes damage can be explained by gender specificities in the levels of a number of biologically active molecules, and in particular sex steroids. Thus, in women, estrogen levels are significantly higher than in men, in whom the predominant steroid is testosterone. That said, estrogens, unlike testosterone, are characterized by numerous cardioprotective effects. Thus, it has been shown that estrogens can have a protective effect against oxidative damage to cardiac myocytes, which plays a significant role in the pathogenesis of atherosclerosis, myocardial ischemia, myocardial hypertrophy and heart failure. The reduction of oxidative damage to cells is associated with a decrease in the formation of reactive oxygen species and an increase in the expression of antioxidant enzymes due to the action of estrogens [69, 70, 71]. In addition, estrogens increase the expression of endothelial nitric oxide synthase, which leads to an increase in the formation of one of the most powerful vasodilators—nitric oxide, and this, in its turn, contributes to greater resistance of the cardiovascular system to coronary vessel spasms (and, accordingly, to a decrease in myocardial blood content), occurring against the background of psycho-emotional stress. Since estrogen production decreases with age in women, cardioprotective effects also decrease, which is expressed by higher levels of cardiospecific troponins in elderly women [50], as reported above in the previous section. Thus, the cardioprotective effects of estrogens can neutralize the degree of damage to cardiac myocytes both under physiological conditions (under stress conditions) and in case of pathological conditions.

Metabolism and renewal of cardiac myocytes [15, 72, 73, 74, 75], which is responsible for the formation of basic serum levels of cardiospecific troponins is regarded as another physiological mechanism for the release of cardiospecific troponins. Taking into account the fact that cardiac myocytes hypertrophy is associated with cardiospecific troponin levels in healthy individuals [11, 76], and in men the myocardial mass (physiological hypertrophy) is bigger than in women [50, 55], the metabolism and renewal of cardiac myocytes can also be considered as a possible mechanism that explains the gender-based variations in the serum levels of cardiospecific troponins. Besides, this mechanism can also explain the gender-based differences in creatine phosphokinase and creatine phosphokinase-MB levels demonstrated in clinical studies [77, 78, 79].

Another important factor contributing to the formation of gender-specific levels of cardiospecific troponins are the analytical parameters of highly sensitive troponin immunoassays. Differences in analytical parameters in different troponin immunoassays may be due to a number of factors, and above all: (1) the use of different anti-cardiospecific troponin antibodies that target different epitopes of cardiospecific troponins, (2) different sensitivity (LoD) of immunoassays, (3) different principles of detection of immunoassays (enzyme immunoassay, immunofluorescence, immunochemiluminescent, radioimmune). Thus, gender differences in the levels of cardiospecific troponins were noted not only between the methods for determining troponin T and troponin I, but also between various high-sensitive methods for determining cardiospecific troponin I [80, 81, 82, 83, 84, 85, 86, 87, 88]. According to the results of a recent meta-analysis, the average difference between the values of cardiospecific troponin I in men and women is 11.0 ng/L (range 7.1–14.9 ng/L) when using a high-sensitive method for determining troponin I (Architect, Abbott Diagnostics) [80]. These gender differences can play a significant diagnostic role in the management of patients with acute coronary syndrome. However, when using a highly sensitive method for determining troponin T (Roche Diagnostics), the average difference between the values for men and women in different reference populations is only 4.6 ng/L (range 1.6–7.6 ng/L), which indicates the insufficient usefulness of the gender approach [80]. Significant differences in gender-specific levels between immunoassays for the determination of cardiospecific troponin I. Therefore, in accordance with recent IFCC recommendations, for optimal use of a gender-oriented approach for the management of patients with acute coronary syndrome, it is necessary to take into account the method of determining cardiospecific troponins [84].

The main factors influencing the formation of gender-specific levels of cardiospecific troponins are summarized in Table 2 (Ref. [68, 69, 70, 71, 72, 73, 74, 75, 80, 81, 82, 83, 84, 85, 86, 87, 88]).

**Table 2.** **The main factors influencing on the formation of gender-specific concentrations of cardiospecific troponins**.

Main factors	Comment	References
Gender differences in the levels of sex hormones (estrogens)	Estrogens have cardioprotective effects due to vasodilation, reduced formation of reactive oxygen species and increased expression of antioxidant molecules	[68, 69, 70, 71]
Gender differences in the metabolism and renewal of cardiac myocytes	Muscle tissue mass, in particular myocardial mass, is associated with the levels of metabolites (biomarkers): creatinine, creatine phosphokinase, creatine phosphokinase-MB, cardiospecific troponins	[72, 73, 74, 75]
Analytical parameters of high-sensitive troponin immunoassays	Different troponin immunoassays have different analytical parameters (LoD, CV, 99th percentile, etc.), so the concentrations in the patient groups will be different	[80, 81, 82, 83, 84, 85, 86, 87, 88]

LoD, limit of detection; CV, coefficient of variation; MB, muscle-brain.

## 6. Conclusions

Thus, introduction of high-sensitive immunochemical tests for determining cardiospecific troponins into clinical practice requires consideration of a number of biological factors of individuals, including gender and age-related characteristics. The optimal level of the 99th percentile is of great importance for the timely diagnostics of acute coronary syndrome and at the same time prevents overdiagnosis of myocardial infarction. Thus, a number of studies have shown that the use of the common 99th percentile can lead to underdiagnosis of acute coronary syndrome in women, since their physiological levels of cardiospecific troponins are much lower. At the same time, the use of the 99th percentile without taking into account the gender factor leads to overdiagnosis of acute coronary syndrome in men, which is due to higher physiological levels of cardiospecific troponins in the blood. According to the IFCC, gender specificities of the 99th percentile are characteristic of most of the existing high-sensitive cardiospecific troponin laboratory tests. Possible mechanisms underlying the gender-based variations in cardiospecific troponin levels are the effects of sex hormones and differences in the myocardial mass. Thus, estrogens have cardioprotective effects, due to which they cause expansion of the coronary vessels, which increases the resistance of cardiac myocytes to physical activities and stressful situations. In addition to the above, estrogens reduce oxidative stress, and thanks to this the damage to cardiac myocytes membranes is limited and the apoptotic mechanisms are suppressed. Apparently, the complex cardioprotective effects of estrogens limit the release of cytoplasmic cardiospecific troponin molecules from the cardiac myocytes into the bloodstream. Further research is needed on the gender specificities of cardiospecific troponin levels: both of clinical (to clarify their significance in the algorithms for diagnosing the acute coronary syndrome) and fundamental nature (to clarify the molecular mechanisms underlying the gender-based variations in the “serum” cardiospecific troponin levels).

## Abbreviations

WHO, the World Health Organization; IFCC, International Federation of Clinical Chemistry; LoD, limit of detection; CV, coefficient of variation; DM, diabetes mellitus; M, men; W, women; EDTA, ethylenediaminetetraacetate.
